# Trust, life satisfaction and health: Population data in mid-size city in the Global South

**DOI:** 10.1016/j.dib.2019.104639

**Published:** 2019-10-10

**Authors:** Lina Martínez

**Affiliations:** Universidad Icesi – POLIS, Cali, Colombia

**Keywords:** Interpersonal and institutional trust, Life satisfaction, Physical and mental health, Social capital, Population survey, Colombia

## Abstract

This paper describes a dataset about institutional and interpersonal trust, life satisfaction and health in Cali, one of the major cities in Colombia, South America. Data was collected with the purpose of monitoring trust levels in the population and to serve as baseline information to monitor changes in population trust after the implementation of government interventions aimed at increasing institutional trust. Data was collected in 2017 with subsequent waves in 2018 and 2019, this manuscript presents data collected in 2017, which corresponds to the data analyzed in the paper related to this manuscript. The information was gathered through a population survey to over 1200 adults' city residents, randomly selected in face to face interviews. Data described also reports information on life satisfaction, physical and mental health, and socioeconomic characteristics. This information is useful for policy making purposes insofar as allow the monitoring of outcomes that are relevant for local and national governments that implement programs that affect trust, subjective well-being and health. Taken as a whole, data also allow to build composite indicators for interpersonal and institutional trust and social capital. This research was fully financed by Universidad Icesi through the Observatorio de Políticas Públicas –POLIS- to monitor citizens' perceptions of a different array of government interventions that affect the outcomes in which data was collected. Measures on trust, life satisfaction, and health follow international measurement standards set by the OEDC and Centers for Disease Control and Prevention to allow international comparisons.

**Table:** Specifications Table

Subject area	Interpersonal and institutional trust, life satisfaction, health, cultural capital
More specific subject area	*Public policy formulation*
Type of data	Text, dummy, and metric variables
How data were acquired	Population survey – Face to face surveys
Data format	Raw
Experimental factors	There is not an experimental component in the data of this manuscript – data was collected through a population survey to randomly selected adults
Experimental features	There was not an experimental component in the data set
Data source location	Cali –Colombia
Data accessibility	By request to: http://www.icesi.edu.co/centros-academicos/polis/
Related research article	Martínez, L. M., Estrada, D., & Prada, S. I. (2019). Mental health, interpersonal trust and subjective well-being in a high violence context. SSM-Population Health, 8, 100423 [1]

**Table:** 

**Value of the data** • There is a high interest in measuring trust. Interpersonal and institutional trust is linked with different social and economic outcomes and there is a wide range of international institutions that seek to make comparative analysis across countries and regions. Trust and its link with different areas are becoming an important factor to measure for policy-making purposes. The data presented in this manuscript is an example of local data that can be used for international comparisons. • Most of the data measuring interpersonal and institutional trust has been collected in the countries in the Global South. Having measures to compare patterns in developing countries, is a stepping stone to build a global dialogue about trust and the many relations it has with government and social outcomes. • Data collected allows international comparisons for analysis aiming at assessing levels of trust in two dimensions: interpersonal and institutional trust. • The data in this dataset also collected widely used measures on life satisfaction, which is a proxy to measure subjective well-being and allows linking trust with overall wellbeing. • Data presented in this analysis also present three health outcomes: general health status, number of days of ill physical health and number of days of ill mental health (30 days' prior to the survey). This a novel feature of data, insofar as there are few data sources that allow connecting trust measures with health outcomes. • There is also relevant sociodemographic information to analyze outcomes of trust, subjective well-being, and health by gender, socioeconomic status, educations, and race/ethnicity. • Taken as a whole, this dataset allows to create composite indicators of interpersonal and institutional trust, subjective well-being, health and build proxies for social capital measurement. By linking these measurements to demographic data, is possible to conduct analysis relevant for policy making analysis at the local level and compare them with international data.

## 1 Data

Data presented in this analysis was collected through face-to-face surveys by trained pollsters in 2017. For data collection, a structured survey was designed (presented as supplementary material) by the Observatorio of Políticas Públicas –POLIS- of Universidad Icesi. POLIS has established a population survey collected annually called CaliBRANDO [2], using this survey, three modules were included: i) life satisfaction; ii) health; and iii) interpersonal and institutional trust. More information about CaliBRANDO and the trust project, is displayed at: https://www.icesi.edu.co/centros-academicos/polis/.

Another important feature of this project is the possibility to link the data with secondary sources of information. Respondents were asked about neighborhood of residency. This variable can be used to be linked with the provision of public services and goods that may be related with the outcome under study.

The module of life satisfaction was constructed using questions from OECD guidelines to measure subjective well-being [3]. In total, 4 questions from the core measure were introduced. One that evaluates the overall life satisfaction, which serves as the primary measure of life satisfaction, and three more that correspond to an affect assessment of this component. Three questions about health were used in this questionnaire, one related to general self-reported health status and two about the number of days of poor physical and mental health (30 days' prior survey collection). Questions for this component comes from the Centers for Disease Control and Prevention [4]. The interpersonal and institutional trust component was build using OECD guidelines to measure trust [5]. Particularly, two sets of questions from the OECD guidelines were introduced: core questions and expectations questions. The survey also included sociodemographic queries (gender, socioeconomic strata, age, and educational attainment). Since all questions included in the survey follow international guidelines, it is possible to conduct comparative analysis.

The questionnaire was piloted 20 times prior implementation and language adjustment were made to correspond to the local context where data was gathered. In 2017, a total of 1217 surveys were collected. Table 1, presents the descriptive statistics of the database. As shown in Table 1, the scale of each question is presented next to the question.

**Table 1:** Descriptive statistics.

**Demographic data**	
Female (%)	50
Average age (years)	39,2
Race/ethnicity	
Minority (%)	64,3
Non-minority (%)	31,6
Average years of educational attainment	11,8
**Life satisfaction component**	
In general, how satisfied are you with your life? (scale 0–10)	8,4
How you felt yesterday – happy (scale 0–10)	8,2
How you felt yesterday – worried (scale 0–10)	3,7
How you felt yesterday – depressed (scale 0–10)	2,1
**Health component**	
Would you say that in general your health is (% excellent – good)	80,6
How many days during the past 30 days was your physical health not good (average days)	2,9
How many days during the past 30 days was your mental health not good (average days)	2,8
**Interpersonal and Institutional trust component**	
In general, how much do you trust most people? (scale 0–10)	4,5
In general, how much do you trust most people you know personally? (scale 0–10)	6,9
Do you think your wallet (or your valuables) would be returned to you if it were found by a neighbor? (% yes)	49,5
Do you think your wallet (or your valuables) would be returned to you if it were found by a stranger? (% yes)	18,6
How much you personally trust – City Council? (scale 0–10)	3
How much you personally trust – The Police? (scale 0–10)	3,8
How much you personally trust – The civil service? (scale 0–10)	2,7
**Total number of observations**	**1237**

Analysis using the data described, are published in an article assessing whether two factors of wellbeing, social capital (interpersonal trust and social networks) and subjective well-being are associated with frequent mental distress and if there are any mediating effects by gender [1].

## 2 Experimental design, materials and methods

The dataset described in this manuscript is representative of the socioeconomic, gender, and racial/ethnic composition of the city with a margin error of 2.8% and a confidence level of 95%. This is a representative sample for the adult population in the city. The sample size of the survey, was estimated using the population reports of the national statics office in Colombia [6]. The survey uses a multistage stratified sampling. The first stage is the selection of 38 points around the city. The second stage defines quotas according to socioeconomic strata, gender and race/ethnicity. The third stage is the random selection of the target population. Raw data for the 2017 data collection is annexed as supplemental material. This data set is anonymized and sensible information is not provided.

**Fig. 1:**
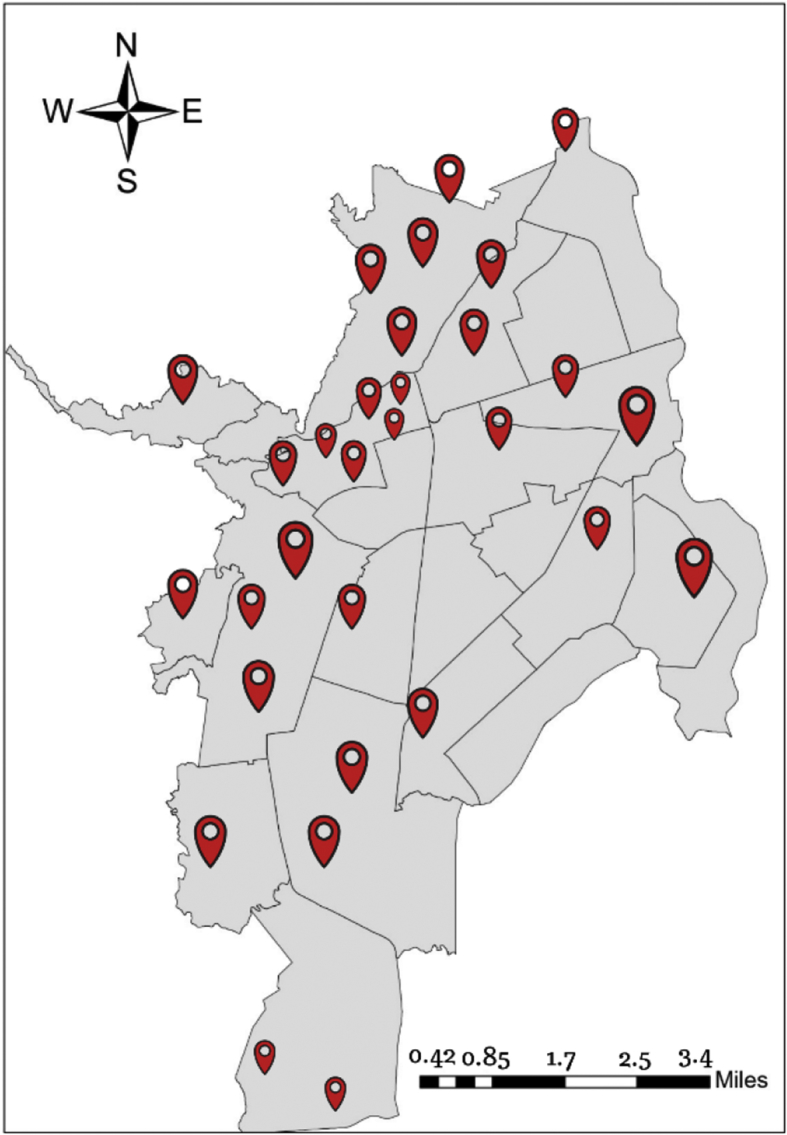
Data collection points in the city.

For field work, pollsters received intensive training about data collection and the language to be used during the survey. In total, 38 points in the city were selected to collect the data. Central points were used for field work instead of household surveys. Generally speaking, through household surveys the major share of the population responding the survey is older, retired or housekeepers. The aim of the study was to collect information representative to the general population and sociodemographic composition of the city. Fieldwork was conducted in parks, shopping malls, bus stations, recreational centers, open commercial areas, and central points in downtown. Where necessary, permission for data collection was solicited prior fieldwork. Fig. 1, presents the city map and the points where surveys were collected.

The survey lasted about 20 minutes. Pollsters approached respondents explaining the purpose of the study and assuring confidentiality, participation in the study was voluntary and respondents received a bookmark with the project information. Field supervisors revised each survey after pollsters filled completely the survey. Each day, a log data entry was updated with information about gender, socioeconomic strata, and race/ethnicity to keep track of the quotas in which the study is representative. Fieldwork lasted about three weeks.
