# Supplementary material for: Trust, life satisfaction and health: Population data in mid-size city in the Global South
Source: Data Brief. 2019 Oct 10;27:104639. doi: 10.1016/j.dib.2019.104639 (PMC6831658; doi:10.1016/j.dib.2019.104639)
Supplement: Multimedia component 2 [file mmc2.pdf]

UNIVERSIDAD ICESI | POLIS | Cali BRANDO | 2017

Aplication date	Day:	Month:	Zone:
Pollster:			Supervisor:

A. The contents of this survey are intended solely for academic purposes. During the process of collecting, processing and analyzing the information you provide, it won´t be possible to identify any respondent. Are you willing to participate in this survey?

1 [ ] Yes   0 [ ] No

DEMOGRAPHIC DATA

1. Gender

1 [ ] Male   2 [ ] Female   3 [ ] Intersex

2. Year of birth:

______________________ [ ]

(Only for adults, people birthed before **1999**)

3. Which is the socio-economic strata of your household?

1 [ ]   4 [ ]
2 [ ]   5 [ ]
3 [ ]   6 [ ]

4. According to your cultural, ethnic group or physical characteristics, how do you recognize yourself?

1 [ ] White   3 [ ] Native   5 [ ] Other
2 [ ] Multi-racial   4 [ ] Black/Afro   6 [ ] None

5. What is the highest education level you reached (even if you didn't finish) and the last grade approved in this level?

*Mark with a X the educational level and write the lasts grade or year in this level

		(1)Level	(2)Year
1	Incomplete primary school		
2	Complete primary school		
3	Imcomple secondary school		
4	Complete secondary school		
5	Technician**		
6	Professional **		
7	Specialization **		
8	Master / Doctorate **		
9	None		NA
10	DK		NA

**Specify semester or years

6. How many years of education your parents attained?

1. Father

__________   99 [ ] DK

2. Mother

__________   99 [ ] DK

SUBJECTIVE WELL-BEING

The following questions are about how you feel, in a scale of 0 to 10. Zero means you have nothing satisfaction and 10 means you have complete satisfaction.

7. In general, how satisfied are you with your life?

Nothing satisfied	0	1	2	3	4	5	6	7	8	9	10	Completely satisfied

The following questions ask about how you felt yesterday on a scale of 0 to 10. Zero means you did not experience the feeling "at all" yesterday while 10 means you experienced the feeling "all of the time" yesterday. I will now read out a list of ways you might have felt yesterday.

Not at all	**0**	**1**	**2**	**3**	**4**	**5**	**6**	**7**	**8**	**9**	**10**	All of the time
**8. How about happy? (WB-A3)**	0	1	2	3	4	5	6	7	8	9	10	
**9. How about worried? (WB-A4)**	0	1	2	3	4	5	6	7	8	9	10	
**10. How about depressed? (WB-A5)**	0	1	2	3	4	5	6	7	8	9	10	

ICESI · POLIS · Cali BRANDO · 2017

HEALTH

11. Would you say that in general your health is

1 [ ] Excelent 2 [ ] Very good 3 [ ] Good

4 [ ] Fair 5 [ ] Poor 6 [ ] DK

12. Now thinking about your physical health, which includes physical illness and injury, for how many days during the past 30 days was your physical health not good?

____
(Number of days)

99 [ ] DK

13. Now thinking about your mental health, which includes stress, depression, and problems with emotions, for how many days during the past 30 days was your mental health not good?

____
(Number of days)

99 [ ] DK

INTERPERSONAL TRUST

And now a general question about trust. On a scale from zero to ten, where zero is not at all and ten is completely.

14. In general, how much do you trust most people?

Not at all	0	1	2	3	4	5	6	7	8	9	10	Completely	88	DK

15. In general, how much do you trust most people you know personally?

Not at all	0	1	2	3	4	5	6	7	8	9	10	Completely	88	DK

In the city or area where you live, imagine that you lost your wallet or something holding your identification or address and it was found by someone else.

16. Do you think your wallet (or your valuables) would be returned to you if it were found by a neighbour?

1 [ ] Yes 2 [ ] No

17. Do you think your wallet (or your valuables) would be returned to you if it were found by a stranger?

1 [ ] Yes 2 [ ] No

INSTITUTIONAL TRUST

The next questions are about whether you have trust in various institutions in Cali. Even if you have had very little or no contact with these institutions, please base your answer on your general impression of these institutions. On a score of 0-10 how much you personally trust each of the institutions I read out. 0 means you do not trust an institution at all, and 10 means you have complete trust.

**17. City Council? (Trust-A3)**	0	1	2	3	4	5	6	7	8	9	10	88	DK
**18. The Police? (Trust-A4)**	0	1	2	3	4	5	6	7	8	9	10	88	DK
**19. The civil service? (Trust-A5)**	0	1	2	3	4	5	6	7	8	9	10	88	DK

The following questions are about your expectations of behaviour from government institutions. In each question, you will be asked whether you think a particular example of behaviour is something that would be expected not to occur at all, or to always occur. Please respond on a scale from 0 to 10 where 0 means not at all and 10 means always.

20. If you complain about bad quality of a public service, how likely is that the problem will be easily resolved? (TRUST-C3)

Not at all	0	1	2	3	4	5	6	7	8	9	10	Always	88	DK

CONTINUATION

21. If a large group of citizens expresses dissatisfaction with the functioning of a public service (e.g. the education, health or justice system), do you think that corrective actions will be taken?

Not at all | 0 | 1 | 2 | 3 | 4 | 5 | 6 | 7 | 8 | 9 | 10 | Always | 88 | DK

22. If a natural disaster occurs, do you think that the provision by government of adequate food, shelter and clothing will be available to people who are affected?

Not at all | 0 | 1 | 2 | 3 | 4 | 5 | 6 | 7 | 8 | 9 | 10 | Always | 88 | DK

23. If money is offered by citizens to government employees in order to speed up administrative procedures, do you think that they will refuse the bribe?

Not at all | 0 | 1 | 2 | 3 | 4 | 5 | 6 | 7 | 8 | 9 | 10 | Always | 88 | DK

24. If a parliamentarian could influence the award of a government contract and receive a bribe in return, do you think that he/she will reject the bribe?

Not at all | 0 | 1 | 2 | 3 | 4 | 5 | 6 | 7 | 8 | 9 | 10 | Always | 88 | DK

25. If a high level politician would be offered a well-paid job in the private sector in exchange for political influence, do you think that he/she will reject the job?

Not at all | 0 | 1 | 2 | 3 | 4 | 5 | 6 | 7 | 8 | 9 | 10 | Always | 88 | DK

26. If you need information about an administrative procedure, do you think that it will be easy to find?

Not at all | 0 | 1 | 2 | 3 | 4 | 5 | 6 | 7 | 8 | 9 | 10 | Always | 88 | DK

27. If a decision affecting your community is to be taken by the local or regional government, how likely is it that you will be consulted?

Not at all | 0 | 1 | 2 | 3 | 4 | 5 | 6 | 7 | 8 | 9 | 10 | Always | 88 | DK

28. If you participate in a public consultation, do you think that your opinion will be considered?

Not at all | 0 | 1 | 2 | 3 | 4 | 5 | 6 | 7 | 8 | 9 | 10 | Always | 88 | DK

29. If a citizen belongs to a social minority (e.g. sexual, racial/ethnic and/or based on nationality), how likely is it that he or she will be treated equally by a government agency?

Not at all | 0 | 1 | 2 | 3 | 4 | 5 | 6 | 7 | 8 | 9 | 10 | Always | 88 | DK

30. If a tax reform is implemented, do you think that the financial burden would be shared fairly across social groups?

Not at all | 0 | 1 | 2 | 3 | 4 | 5 | 6 | 7 | 8 | 9 | 10 | Always | 88 | DK
